# Dynamics of Hepatitis B Virus Quasispecies in Association with Nucleos(t)ide Analogue Treatment Determined by Ultra-Deep Sequencing

**DOI:** 10.1371/journal.pone.0035052

**Published:** 2012-04-16

**Authors:** Norihiro Nishijima, Hiroyuki Marusawa, Yoshihide Ueda, Ken Takahashi, Akihiro Nasu, Yukio Osaki, Tadayuki Kou, Shujiro Yazumi, Takeshi Fujiwara, Soken Tsuchiya, Kazuharu Shimizu, Shinji Uemoto, Tsutomu Chiba

**Affiliations:** 1 Department of Gastroenterology and Hepatology, Graduate School of Medicine, Kyoto University, Kyoto, Japan; 2 Department of Gastroenterology and Hepatology, Osaka Red Cross Hospital, Osaka, Japan; 3 Department of Gastroenterology and Hepatology, Tazuke Kofukai Medical Research Institute, Kitano Hospital, Osaka, Japan; 4 Department of Nanobio Drug Discovery, Graduate School of Pharmaceutical Sciences, Kyoto University, Kyoto, Japan; 5 Department of Surgery, Graduate School of Medicine, Kyoto University, Kyoto, Japan; Singapore Institute for Clinical Sciences, Singapore

## Abstract

**Background and Aims:**

Although the advent of ultra-deep sequencing technology allows for the analysis of heretofore-undetectable minor viral mutants, a limited amount of information is currently available regarding the clinical implications of hepatitis B virus (HBV) genomic heterogeneity.

**Methods:**

To characterize the HBV genetic heterogeneity in association with anti-viral therapy, we performed ultra-deep sequencing of full-genome HBV in the liver and serum of 19 patients with chronic viral infection, including 14 therapy-naïve and 5 nucleos(t)ide analogue(NA)-treated cases.

**Results:**

Most genomic changes observed in viral variants were single base substitutions and were widely distributed throughout the HBV genome. Four of eight (50%) chronic therapy-naïve HBeAg-negative patients showed a relatively low prevalence of the G1896A pre-core (pre-C) mutant in the liver tissues, suggesting that other mutations were involved in their HBeAg seroconversion. Interestingly, liver tissues in 4 of 5 (80%) of the chronic NA-treated anti-HBe-positive cases had extremely low levels of the G1896A pre-C mutant (0.0%, 0.0%, 0.1%, and 1.1%), suggesting the high sensitivity of the G1896A pre-C mutant to NA. Moreover, various abundances of clones resistant to NA were common in both the liver and serum of treatment-naïve patients, and the proportion of M204VI mutants resistant to lamivudine and entecavir expanded in response to entecavir treatment in the serum of 35.7% (5/14) of patients, suggesting the putative risk of developing drug resistance to NA.

**Conclusion:**

Our findings illustrate the strong advantage of deep sequencing on viral genome as a tool for dissecting the pathophysiology of HBV infection.

## Introduction

Hepatitis B virus (HBV) is a non-cytopathic DNA virus that infects approximately 350 million people worldwide and is a main cause of liver-related morbidity and mortality [1]–[3]. The absence of viral-encoded RNA-dependent DNA polymerase proofreading capacity coupled with the extremely high rate of HBV replication yields the potential to rapidly generate mutations at each nucleotide position within the entire genome [4]. Accordingly, a highly characteristic nature of HBV infection is the remarkable genetic heterogeneity at the inter- and intra- patient level. The latter case of variability as a population of closely-related but nonidentical genomes is referred to as viral quasispecies [5], [6]. It is well recognized that such mutations may have important implications regarding the pathogenesis of viral disease. For example, in chronic infection, G to A point mutation at nucleotide (nt) 1896 in the pre-core (pre-C) region as well as A1762T and G1764A mutations in the core-promoter region are highly associated with HBeAg seroconversion that in general results in the low levels of viremia and consequent clinical cure [7]–[9]. In contrast, acute infection with the G1896A pre-C mutant represents a high risk for fulminant hepatic failure [10], [11]. Although these facts clearly illustrate the clinical implications of certain viral mutation, increasing evidence strongly suggests that the viral genetic heterogeneity is more complicated than previously thought [12], [13].

The major goals of antiviral therapy in patients with HBV infection are to prevent the progression of liver disease and inhibit the development of hepatocellular carcinoma [14]. Oral nucleos(t)ide analogue (NA) have revolutionized the management of HBV infection, and five such antiviral drugs, including lamivudine, adefovir, entecavir, tenofovir, and telbivudine, are currently approved medications [15], [16]. These agents are well-tolerated, very effective at suppressing viral replication, and safe, but one of the major problems of NA therapy is that long-term use of these drugs frequently causes the emergence of antiviral drug-resistant HBV due to substitutions at specific sites in the viral genome sequences, which often negates the benefits of therapy and is associated with hepatitis flares and death [16], [17]. It is unclear whether viral clones with antiviral resistance emerge after the administration of antiviral therapy or widely preexist among treatment-naïve patients.

There has been a recent advance in DNA sequencing technology [18]. The ultra-deep sequencers allow for massively parallel amplification and detection of sequences of hundreds of thousands of individual molecules. We recently demonstrated the usefulness of ultra-deep sequencing technology to unveil the massive genetic heterogeneity of hepatitis C virus (HCV) in association with treatment response to antiviral therapy [19]. On the other hand, there are a few published studies in which this technology was used to characterize genetic HBV sequence variations [20]–[22]. Margeridon-Thermet et al reported that the 454 Life Science GS20 sequencing platform provided higher sensitivity for detecting drug-resistant HBV mutations in the serum of patients treated with nucleoside and nucleotide reverse-transcriptase inhibitors [20]. Solmone et al also reported the strong advantage conferred by the same platform to detect minor variants in the serum of patients with chronic HBV infection [21]. Although in these previous studies low-abundant drug-resistant variants were successfully detected, the analyses were focused on the reverse-transcriptase region of circulating HBV in the serum and thus the whole picture of HBV genetic heterogeneity as well as the *in vivo* dynamics of HBV drug resistant variants in response to anti-viral treatment remains to be clarified. Moreover, intrahepatic viral heterogeneity in patients that achieved the clearance of circulating HBV is largely unknown.

By taking the advantage of an abundance of genetic information obtained by utilizing the Illumina Genome Analyzer II (Illumina, San Diego, CA) as a platform of ultra-deep sequencing, we determined the whole HBV sequence in the liver and serum of patients with chronic HBV infection to evaluate viral quasispecies characteristics. Moreover, we investigated the prevalence of rare drug-resistant HBV variants as well as detailed dynamic changes in the viral genetic heterogeneity in association with NA administration. Based on the abundant genetic information obtained by ultra-deep sequencing, we clarified the precise prevalence of HBV clones with G1896A pre-C mutations in association with HBe serostatus in chronically infected patients with or without NA treatment. We also detected a variety of minor drug-resistant clones in treatment-naïve patients and their dynamic changes in response to entecavir administration, demonstrating the potential clinical significance of naturally-occurring drug-resistant mutations.

## Materials and Methods

### Ethics Statement

The Kyoto University ethics committee approved the study, and written informed consent for participation in this study was obtained from all patients. The study was conducted in accordance with the principles of the Declaration of Helsinki.

### Patients

The liver tissues of 19 Japanese patients that underwent living-donor liver transplantation at Kyoto University due to HBV-related liver disease were available for viral genome analyses. These individuals included 13 men and 6 women, aged 41 to 69 years (median, 55.2 years) and all but one were infected with genotype C viruses. Participants comprised 19 patients with liver cirrhosis caused by chronic HBV infection, including 14 antiviral therapy-naïve cases (chronic-naïve cases) and 5 cases receiving NA treatment, with either lamivudine or entecavir (chronic-NA cases) (Table 1). Serum HBV DNA levels were significantly higher in chronic-naïve cases than in chronic NA cases (median serum HBV DNA levels were 5.6, and <2.6 log copies/ml, respectively, Table 1). Liver tissue samples were obtained at the time of transplantation, frozen immediately, and stored at −80°C until use. Serologic analyses of HBV markers, including hepatitis B surface antigen (HBsAg), antibodies to HBsAg, anti-HBc, HBeAg, and antibodies to HBeAg, were determined by enzyme immunoassay kits as described previously [23]. HBV DNA in the serum before transplantation was examined using a polymerase chain reaction (PCR) assay (Amplicor HBV Monitor, Roche, Branchburg, NJ). To examine the dynamics of viral quasispecies in response to anti-HBV therapy, paired serum samples of 14 treatment-naïve patients before and after administration of daily entecavir (0.5 mg/day) were subjected to further analyses on viral genome.

**Table 1:** Characteristics of patients with chronic HBV infection analyzed in this study.

	Chronic-naïve (N = 14)	Chronic-NA (N = 5)
Age†	55.5 (41–69)	55.0 (49–68)
Sex (male/female)	9/5	4/1
Alanine aminotransaminase (IU/l)†	41 (10–74)	30 (15–65)
Total bilirubin (mg/dl)†	0.9 (0.5–31.1)	1.7 (0.6–4.5)
Platelet count (×10^4^/mm^3^)†	12.7 (3.3–27.6)	5.1 (3.6–11.3)
HBV genotype		
B	1	0
C	13	5
Viral load (log copies /ml)†	5.6 (<2.6–8.8)*	<2.6 (<2.6–5.3)*
HBe-serostatus (HBeAg+/HBeAb+)	8/6	0/5
Fibrosis		
F0–F2	6	0
F3–F4	8	5
Activity		
A0–A1	7	3
A2–A3	7	2

† Values are median (range).

* P = 0.042.

### Direct population Sanger sequencing

DNA was extracted from the liver tissue and serum using a DNeasy Blood & Tissue Kit (Qiagen, Tokyo, Japan). To define the consensus reference sequences of HBV in each clinical specimen, all samples were first subjected to direct population Sanger sequencing using the Applied Biosystems 3500 Genetic Analyzer (Applied Biosystems, Foster City, CA). Oligonucleotide primers for the HBV genome were designed to specifically amplify whole viral sequences as two overlapping fragments using the sense primer 169_F and antisense primer 2847_R to yield a 2679-bp amplicon (amplicon 1), and the sense primer 685_F and antisense primer 443_R to yield a 2974-bp amplicon (amplicon 2; Table S1). HBV sequences were amplified using Phusion High-Fidelity DNA polymerase (FINZYMES, Espoo, Finland). All amplified PCR products were purified using the QIAquick Gel Extraction kit (Qiagen) after agarose gel electrophoresis and used for direct sequencing. The serum of a healthy HBV DNA-negative volunteer was used as a negative control.

### Viral genome sequencing by massively-parallel sequencing

Massively-parallel sequencing with multiplexed tags was performed using the Illumina Genome Analyzer II as described [19]. The end-repair of DNA fragments, addition of adenine to the 3′ ends of DNA fragments, adaptor ligation, and PCR amplification by Illumina PCR primers were performed as described previously [24]. Briefly, the viral genome sequences were amplified by high-fidelity PCR using oligonucleotide primers as described above, sheared by nebulization using 32 psi N2 for 8 min, and then the sheared fragments were purified and concentrated using a QIAquick PCR purification Kit (Qiagen). Nucleotide overhangs resulting from fragmentation were then converted into blunt ends using T4 DNA polymerase and Klenow enzymes, followed by the addition of terminal 3′ A-residues. An adaptor containing unique 6-bp tags, such as “ATCACG” and “CGATGT” (Multiplexing Sample Preparation Oligonucleotide Kit, Illumina), was then ligated to each fragment using DNA ligase. We then performed agarose gel electrophoresis of adaptor-ligated DNAs and excised bands from the gel to produce libraries with insert sizes ranging from 200 to 350 bp. These libraries were amplified independently using a minimal PCR amplification step of 18 cycles by Illumina PCR primers with Phusion High-Fidelity DNA polymerase. The DNA fragments were then purified with a MinElute PCR Purification Kit (Qiagen), followed by quantification using the NanoDrop 2000C (Thermo Fisher Scientific, Waltham, MA) to make a working concentration of 10 nM. Cluster generation and sequencing was performed for 64 cycles on the Illumina Genome Analyzer II according to the manufacturer's instructions. The obtained images were analyzed and base-called using GA pipeline software version 1.4 with the default settings provided by Illumina.

### Genome Analyzer sequence data analysis

Using the high performance alignment software “NextGene“ (SoftGenetics, State College, PA), the 64 base-pair reads obtained from the Genome Analyzer II were aligned with the reference sequences of 3215 bp that were determined by direct population Sanger sequencing of each clinical specimen. Reads with 90% or more bases matching a particular position of the reference sequences were aligned. Furthermore, two quality filters were used for sequencing reads: the reads with a median quality score of more than 30 and no more than 3 uncalled nucleotides were allowed anywhere in the 64 bases. Only sequences that passed the quality filters, rather than raw sequences, were analyzed and each position of the viral genome was assigned a coverage depth, representing the number of times the nucleotide position was sequenced.

### Allele-specific quantitative real-time PCR and semiquantitative PCR to determine the relative proportion of G1896A pre-C mutant

To determine the relative proportion of the G1896A pre-C mutant, allele-specific quantitative real-time PCR was performed based on the previously described method [25], [26]. Oligonucleotide primers were designed individually to amplify the pre-C region of wild-type and the G1896A pre-C mutant HBV. Three primers were used for this protocol, two allele-specific sense primers, 1896WT_F (for wild-type) and 1896MT_F (for the G1896A pre-C mutant), and one common antisense primer, 2037_R (Table S1). Quantification of wild-type and the G1896A pre-C mutant was individually performed by real-time PCR using a Light Cycler 480 and Fast Start Universal SYBR Master (Roche, Mannheim, Germany) [27]. The relative proportion of the G1896A pre-C mutant was determined to calculate the G1896A pre-C mutant/total HBV ratios. Performance of this assay was tested using mixtures of two previously described plasmids, pcDNA3-HBV-wt#1 and pcDNA3-HBV-G1896A pre-C mutant [28]. Semiquantitative PCR was performed using primers described above, then agarose gel electrophoresis was performed.

### Statistical analysis

Results are expressed as mean or median, and range. Pretreatment values were compared using the Mann-Whitney U-test or the Kruskal Wallis H-test. *P* values less than 0.05 were considered statistically significant.

The viral quasispecies characteristics were evaluated by analyzing the genetic complexity based on the number of different sequences present in the population. Genetic complexity for each site was determined by calculating the Shannon entropy using the following formula:
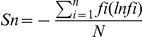
where *n* is the number of different species identified, *fi* is the observed frequency of a particular variant in the quasispecies, and *N* is the total number of clones analyzed [12], [13]. The mean viral complexity in each sample was determined by calculating the total amounts of the Shannon entropy at each nucleotide position divided by the total nucleotide number (e.g., 3215 bases) of each HBV genome sequence.

### Nucleotide sequence accession number

All sequence reads have been deposit in DNA Data Bank of Japan Sequence Read Archive (http://www.ddbj.nig.ac.jp/index-e.html) under accession number DRA000435.

## Results

### Validation of multiplex ultra-deep sequencing of the HBV genome

To differentiate true mutations from sequencing errors in the determined sequences, we first generated viral sequence data from the expression plasmid, pcDNA3-HBV-wt#1, encoding wild-type genotype C HBV genome sequences [28]. For this purpose, we determined the PCR-amplified HBV sequences derived from the expression plasmid using high-fidelity Taq polymerase to take the PCR-induced errors as well as sequencing errors into consideration. Viral sequences determined by the conventional Sanger method were used as reference sequences for aligning the amplicons obtained by ultra-deep sequencing. Three repeated ultra-deep sequencing generated a mean of 77,663 filtered reads, corresponding to a mean coverage of 38,234 fold at each nucleotide site (Table S2). Errors comprised insertions (0.00003%), deletions (0.00135%), and nucleotide mismatches (0.037%). The mean overall error rate was 0.034% (distribution of per-nucleotide error rate ranged from 0 to 0.13%) for the three control experiments, reflecting the error introduced by high-fidelity PCR amplification and by multiplex ultra-deep sequencing that remained after filtering out problematic sequences. We also confirmed that multiplex ultra-deep sequencing with and without the high-fidelity PCR amplification with HBV-specific primer sets showed no significant differences in the error rates on the viral sequence data (mean error rate 0.034% vs 0.043%). Accordingly, we defined the cut-off value in its current platform as 0.3%, a value nearly 1 log above the mean overall error rate.

Next, we performed additional control experiments to verify the detectability of the low abundant mutations that presented at a frequency of less than 0.3%. For this purpose, we introduced expression plasmids with a single-point mutation within that encoding a wild-type viral sequence with a ratio of 1∶1000 and assessed the sensitivity and accuracy of quantification using high-fidelity PCR amplification followed by multiplex ultra-deep sequencing in association with the different coverage numbers (Table S3). Repeated control experiments revealed that the threshold for detecting low-abundant mutations at an input ratio of 0.10% among the wild-type sequences ranged between 0.11% and 0.24%, indicating that there was no significant difference in the detection rate or error rates under the different coverage conditions. Based on these results, the accuracy of ultra-deep sequencing in its current platform for detecting low-level viral mutations was considered to be greater than 0.30%.

### Viral complexity of the HBV quasispecies in association with clinical status

To clarify HBV quasispecies in association with clinical status, we performed multiplex ultra-deep sequencing and determined the HBV full-genome sequences in the liver and serum with chronic HBV infection. First, we compared the sequences of the viral genome determined in the liver tissue with those in the serum and found no significant differences in the viral population between the liver and serum of the same individual. Indeed, the pattern and distribution of genetic heterogeneity of the viral nucleotide sequences in the liver tissue were similar to those observed in the serum of the same patient (Figure S1), suggesting that a similar pattern of viral heterogeneity was maintained in the liver and serum of patients with chronic HBV infection.

**Figure 1:**
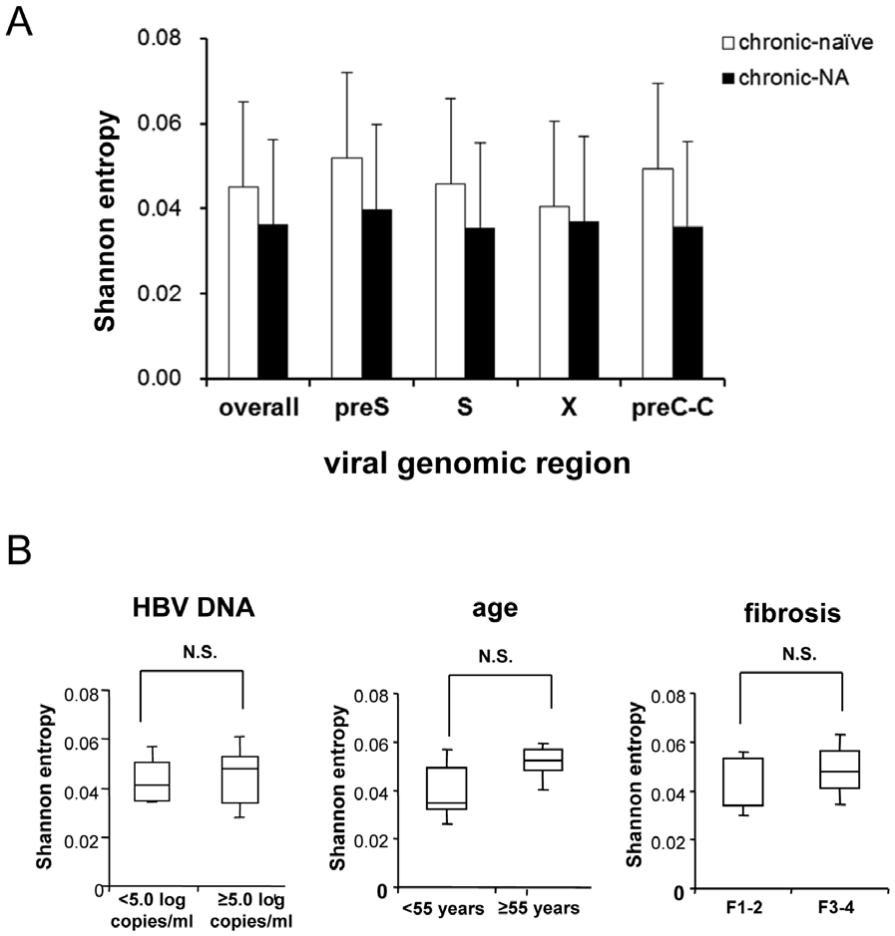
Viral complexity of the HBV quasispecies in association with clinical status. (A) The Shannon entropy values for each viral genomic region were determined in the liver of chronic-naïve and chronic-NA cases. (B) Among the chronic-naïve cases, the Shannon entropy values are shown for patients with serum HBV DNA levels less than 5.0 log copies/ml (<5.0) and greater than 5.0 log copies/ml (≥5.0) (left panel), patients under the age of 55 years (<55) and over the age of 55 (≥55) (middle panel), and patients with low (F1–2) and high (F3–4) liver fibrosis levels (right panel). preS: pre-surface, preC-C: precore∼core N.S.: not significant.

Next, we compared the viral heterogeneity in the liver of chronic-naïve and chronic-NA cases. A mean of 5,962,996 bp nucleotides in chronic-naïve cases and 4,866,783 bp nucleotides in chronic-NA cases were mapped onto the reference sequences, and an overall average coverage depth of 1,855 and 1.514 was achieved for each nucleotide site of the HBV sequences, respectively (Table 2). The frequencies of mutated positions and altered sequence variations detected in each viral genomic region are summarized in Table 2. The overall mutation frequency of the total viral genomic sequences was determined to be 0.87% in chronic-naïve cases and 0.69% in chronic-NA cases. Most genomic changes observed in viral variants were single base substitutions, and the genetic heterogeneity of the viral nucleotide sequences was equally observed throughout the individual viral genetic regions, including the pre-surface (preS), S, pre-core∼core (preC-C), and X (Table 2). Consistent with the findings obtained from the viral mutation analyses, the overall viral complexity determined by the Shannon entropy value was 0.047 in chronic-naïve and 0.036 in chronic-NA cases, and the viral complexity was equally observed throughout the individual viral genetic region (Figure 1A). Among chronic-naïve cases, we observed no significant differences in the viral complexity in HBV DNA level, age, or degree of fibrosis (Figure 1B).

**Table 2:** The frequency of mutation rate and the Shannon entropy in each viral genome region.

	Liver	
	Chronic-naïve (N = 14)	Chronic-NA (N = 5)
Average aligned reads	93,172	76,043
Average aligned nucleotides	5,962,996	4,866,783
Average coverage	1,855	1,514
Mutation rate (%)		
Overall	0.87	0.69
preS	0.92	0.81
S	0.96	0.71
preC-C	1.05	0.72
X	0.63	0.61
Shannon entropy	0.047	0.036

Mutation rate (%): the ratio of total different nucleotides from the reference sequence to total aligned nucleotides.

preS: pre-surface, preC-C: pre-core∼core.

### High sensitivity of the G1896A pre-C mutant to nucleos(t)ide analogues

Emergence of G1896A mutation in the pre-C region, and A1762T and G1764A mutations in the core-promoter region is well known to be associated with HBe-seroconversion [7]–[9]. We then evaluated the prevalence of these three mutations in the chronically HBV-infected liver, in association with HBe serologic status and the NA treatment history. In chronic-naive cases, 6 and 8 patients showed the pre- and post- HBeAg seroconversion status, respectively (Table 3). The mean prevalence of the G1896A pre-C mutant in HBeAg-positive cases was lower than that in the HBeAg-negative cases (27.4% and 46.5%, respectively). Importantly, however, 4 of 8 HBeAg-negative cases showed a relatively low prevalence of the G1896A pre-C mutant (Liver #8, #12, #13, #14), and all but one case (Liver #10) showed a high prevalence of the A1762T and G1764A mutations, irrespective of HBe serologic status and NA treatment history (Table 3). These findings suggested that other mutations except G1896A, A1762T and G1764A were also involved in the HBeAg seroconversion status. Notably, liver tissues of all but one (Liver #17) chronic-NA cases showed extremely low levels of the G1896A pre-C mutant (0.0, 0.0, 0.1, and 1.1%), suggesting the high sensitivity of the G1896A pre-C mutant to NA (Table 3).

**Table 3:** The prevalence of G1896A mutation in the pre-C region, and A1762T and G1764A mutations in the core-promoter region in the liver of patients chronically infected with HBV.

			Mutation Frequency					
	HBeAg/HBeAb	NA (duration of treatment)	G1896A (Pre C)		A1762T (CP)		G1764A (CP)	
**Chronic-naive**								
Liver #1	+/−	-	640/1652	(38.7)	1647/1941	(84.9)	1683/1979	(85.0)
Liver #2	+/−	-	9/596	(1.5)	682/687	(99.3)	683/689	(99.1)
Liver #3	+/−	-	273/672	(40.6)	767/769	(99.7)	757/760	(99.6)
Liver #4	+/−	-	204/701	(29.1)	610/625	(97.6)	602/621	(96.9)
Liver #5	+/−	-	27/152	(17.8)	249/250	(99.6)	245/248	(98.8)
Liver #6	+/−	-	228/621	(36.7)	727/729	(99.7)	743/744	(99.9)
Liver #7	−/+	-	740/1193	(62.0)	1908/1913	(99.7)	1888/1913	(98.7)
Liver #8	−/+	-	111/1892	(5.9)	2321/2325	(99.8)	2335/2339	(99.8)
Liver #9	−/+	-	10935/10944	(99.9)	12019/12032	(99.9)	12163/12170	(99.9)
Liver #10	−/+	-	4554/4593	(99.2)	1/5191	(0)	4/5188	(0.1)
Liver #11	−/+	-	811/921	(88.1)	1234/1236	(99.8)	1226/1228	(99.8)
Liver #12	−/+	-	93/1265	(7.4)	1234/1234	(100)	1228/1229	(99.9)
Liver #13	−/+	-	83/877	(9.5)	1465/1529	(95.8)	1485/1549	(95.9)
Liver #14	−/+	-	0/717	(0)	1078/1410	(76.5)	1089/1414	(77.0)
**Chronic-NA**								
Liver #15	−/+	LAM (156w)	0/390	(0)	441/453	(97.4)	435/448	(97.1)
Liver #16	−/+	ETV (1w)	0/1399	(0)	1624/1632	(99.5)	1625/1630	(99.7)
Liver #17	−/+	LAM (144w)	345/816	(42.3)	988/991	(99.7)	994/994	(100)
Liver #18	−/+	LAM (98w)	2/3963	(0.1)	1015/1188	(85.4)	1190/1194	(99.7)
Liver #19	−/+	LAM (11w)	48/4214	(1.1)	3438/3456	(99.5)	3446/3462	(99.5)

Values in parenthesis show mutation frequency (%): the ratio of total mutant clones to total aligned coverage at each nucleotide sites.

NA: nucleotide analogue, pre C: precore, CP: core promoter, LAM: lamivudine, ETV: entecavir.

**Figure 2:**
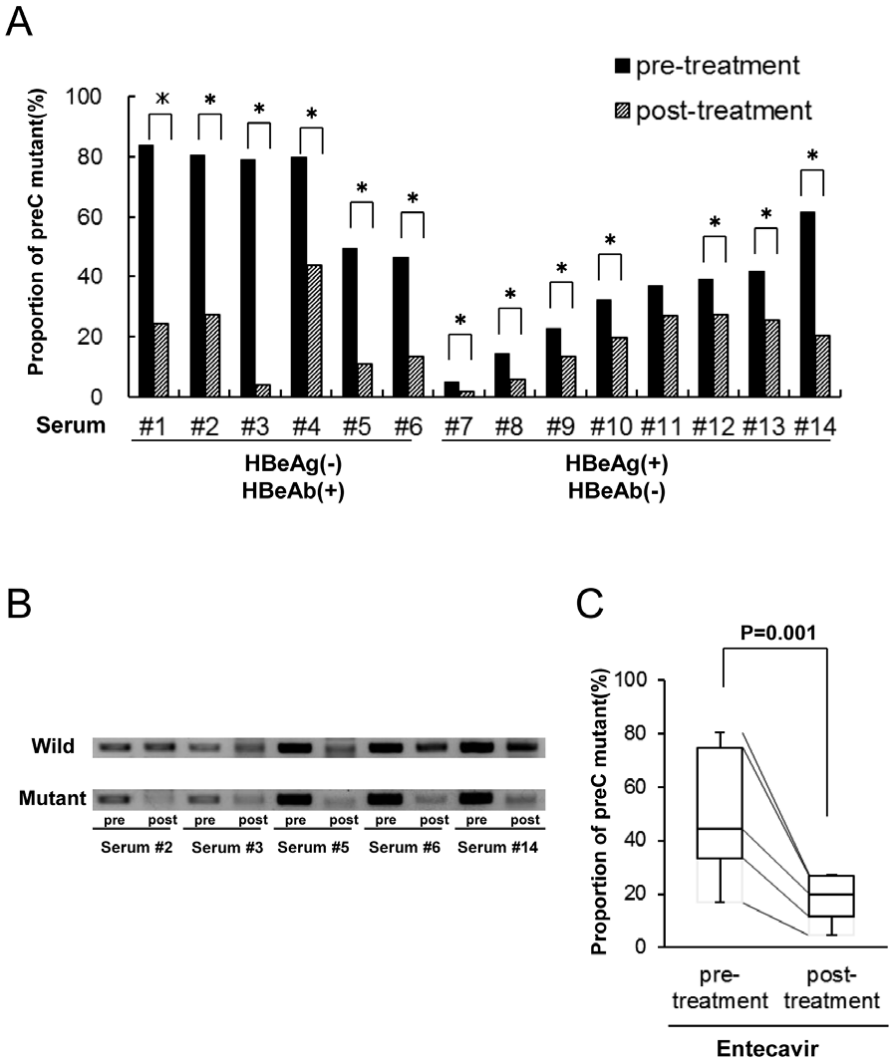
The reduction in the relative proportion of the G1896A pre-C mutant clones after entecavir administration. (A) The relative proportion of the G1896A pre-C mutant was determined in the serum of treatment-naïve patients pre- and post-entecavir administration using quantitative real-time PCR. Serum #1∼6 were HBeAg-negative and HBeAb-positive, and Serum #7∼14 were HBeAg-positive and HBeAb-negative before treatment. *: p<0.05 (B) Semiquantitative PCR analysis was performed using primers specific to the wild-type (upper panel) or G1896A pre-C mutant (lower panel) pre- and post-entecavir administration. A representative result from 5 cases is shown. (C) The relative proportion of the G1896A pre-C mutant was compared in 14 treatment-naïve patients between pre- and post-entecavir administration.

To confirm the difference of the sensitivity to NA between the wild-type and the G1896A pre-C mutant, we examined the dynamic changes of the relative proportion of the G1896A pre-C mutant in the serum of 14 treatment-naïve patients before and after entecavir administration. Consistent with the findings obtained by ultra-deep sequencing, quantitative real-time PCR revealed that entecavir administration significantly reduced the proportion of the G1896A pre-C mutant in 13 of 14 cases (92.9%) irrespective of their HBeAg serostatus, while the G1896A pre-C mutant were detectable in substantial proportion before treatment in all cases (Figure 2A, 2B and 2C; p = 0.001). These results further support the findings that HBV clones comprising the G1896A mutation were more sensitive to NA than those with wild-type sequences.

### Prevalence of drug-resistant HBV clones in the liver of treatment-naïve patients

Increasing evidence suggests that drug-resistant viral mutants can be detected in the serum of treatment-naïve patients with chronic HBV infection [20], [21]. Thus, we next determined the actual prevalence of spontaneously-developed drug-resistant mutants in chronically-infected liver of treatment-naïve patients to evaluate whether NA treatment potentiates the expansion of drug-resistant clones. The drug-resistant mutations examined included two mutations resistant to lamivudine and entecavir, four mutations resistant to entecavir, and three mutations resistant to adefovir [16], [17]. Based on the detection rate of the low-level viral clones determined by the control experiments, we identified the drug-resistant mutants present in each specimen at a frequency of more than 0.3% among the total viral clones. Based on these criteria, at least one resistant mutation was detected in the liver of all of the chronic-naïve cases with chronic HBV infection (Table 4). The prevalence of the 9 drug-resistant mutations detected by ultra-deep sequencing in 14 chronic-naïve cases ranged from 0.3% to 30.0%, indicating that the proportion of resistant mutations substantially differed in each case. The most commonly detected mutation was M204VI (9 cases) and M250VI (11 cases), which were resistant to lamivudine and entecavir, and entecavir, respectively. Other mutations resistant to adefovir were detected in 7 (50.0%) and 3 (21.4%) cases at A181TV and N236T, respectively (Table 4).

**Table 4:** The prevalence of the 9 drug-resistant mutations detected by ultra-deep sequencing derived from liver tissue.

	M204V/I		L180M		T184S/A/I/ L/G/C/M		S202C/G/I		I169T	
Drugs	LAM/ETV		LAM/ETV		ETV		ETV		ETV	
**Chronic-naive**										
Liver #1	**27/5421**	**(0.5%)**	2/3694	(-)	9/3886	(-)	5/5613	(-)	5/3784	(-)
Liver #2	**35/5344**	**(0.7%)**	0/538	(-)	1/563	(-)	17/6340	(-)	0/512	(-)
Liver #3	**13/1363**	**(1.0%)**	0/304	(-)	1/358	(-)	1/1379	(-)	0/264	(-)
Liver #4	11/5113	(-)	0/556	(-)	**2/547**	**(0.4%)**	11/5133	(-)	0/639	(-)
Liver #5	**2/117**	**(1.1%)**	0/409	(-)	1/380	(-)	1/189	(-)	1/474	(-)
Liver #6	12/8451	(-)	0/309	(-)	0/328	(-)	22/8457	(-)	0/334	(-)
Liver #7	**10/3098**	**(0.3%)**	1/1547	(-)	3/1477	(-)	8/3161	(-)	0/1621	(-)
Liver #8	**13/2442**	**(0.5%)**	1/2378	(-)	6/2312	(-)	1/2564	(-)	1/2507	(-)
Liver #9	**67/13879**	**(0.5%)**	2/5443	(-)	2/5107	(-)	6/13804	(-)	0/5650	(-)
Liver #10	16/7400	(-)	0/3524	(-)	3/3283	(-)	5/7113	(-)	0/3492	(-)
Liver #11	0/412	(-)	1/1328	(-)	**1/295**	**(0.3%)**	0/425	(-)	3/4729	(-)
Liver #12	**4/1098**	**(0.4%)**	1/1389	(-)	0/1272	(-)	2/1102	(-)	0/1544	(-)
Liver #13	**8/2476**	**(0.3%)**	1/2192	(-)	3/2085	(-)	4/2529	(-)	4/5029	(-)
Liver #14	5/3713	(-)	0/2009	(-)	4/1925	(-)	2/3820	(-)	5/3784	(-)
**Chronic-NA**										
Liver #15	0/339	(-)	0/49	(-)	0/49	(-)	0/338	(-)	0/40	(-)
Liver #16	**28/7278**	**(0.4%)**	0/4403	(-)	6/4053	(-)	14/7556	(-)	6/6084	(-)
Liver #17	**177/945**	**(18.7%)**	0/1059	(-)	0/1009	(-)	0/945	(-)	0/1051	(-)
Liver #18	**13/2655**	**(0.5%)**	0/1239	(-)	0/1185	(-)	**10/2708**	**(0.4%)**	0/1332	(-)
Liver #19	**80/6795**	**(1.2%)**	0/3168	(-)	2/2971	(-)	3/6734	(-)	0/3384	(-)

	M250V/I		A181T/V		N236T		P237H	
Drugs	ETV		ADV		ADV		ADV	
**Chronic-naive**								
Liver #1	**23/2719**	**(0.9%)**	10/3755	(-)	4/4210	(-)	2/4139	(-)
Liver #2	**9/2079**	**(0.4%)**	**2/549**	**(0.4%)**	1/1144	(-)	1/1188	(-)
Liver #3	**10/1699**	**(0.6%)**	**1/298**	**(0.3%)**	3/1636	(-)	1/1666	(-)
Liver #4	**3/388**	**(0.8%)**	**3/549**	**(0.5%)**	0/560	(-)	0/533	(-)
Liver #5	**2/91**	**(2.2%)**	1/409	(-)	0/55	(-)	0/60	(-)
Liver #6	0/214	(-)	**6/305**	**(2.0%)**	**1/294**	**(0.3%)**	0/257	(-)
Liver #7	**7/1289**	**(0.5%)**	4/1531	(-)	**24/2738**	**(0.9%)**	1/2692	(-)
Liver #8	2/1117	(-)	**689/2336**	**(29.5%)**	2/1713	(-)	0/1639	(-)
Liver #9	**27/7325**	**(0.4%)**	**38/5334**	**(0.7%)**	1/6607	(-)	4/6702	(-)
Liver #10	**12/3815**	**(0.3%)**	0/3454	(-)	**13/3245**	**(0.4%)**	2/3272	(-)
Liver #11	**1/199**	**(0.5%)**	1/972	(-)	0/251	(-)	0/251	(-)
Liver #12	**2/672**	**(0.3%)**	**408/1362**	**(30.0%)**	0/598	(-)	0/597	(-)
Liver #13	1/947	(-)	2/2160	(-)	0/1406	(-)	1/1374	(-)
Liver #14	**23/2719**	**(0.9%)**	10/3755	(-)	4/4210	(-)	2/4139	(-)
**Chronic-NA**								
Liver #15	**1/303**	**(0.3%)**	**2/49**	**(4.1%)**	0/377	(-)	0/384	(-)
Liver #16	1/922	(-)	0/4403	(-)	1/1597	(-)	3/1572	(-)
Liver #17	0/755	(-)	1/1050	(-)	0/698	(-)	**145/698**	**(20.8%)**
Liver #18	1/1464	(-)	2/1206	(-)	0/3156	(-)	0/3107	(-)
Liver #19	8/3834	(-)	**16/3128**	**(0.5%)**	0/3372	(-)	0/3428	(-)

(-): mutant clones less than 0.3% among total clones at each nucleotide sites.

LAM: lamivudine, ADV: adefovir, ETV: entecavir.

Nine (64.2%) chronic-naïve cases possessed the M204VI mutants in their liver tissues and the proportion of mutant clones among the totally infected viruses ranged from 0.3% to 1.1% among the M204VI mutant-positive patients. In chronic-NA cases, 4 of 5 (80.0%) liver tissues harbored the M204VI mutants with the proportion among the totally infected viruses ranging from 0.4% to 18.7% (Table 4), while the mean serum HBV DNA was suppressed below 2.6 log copies/ml (Table 1). These results suggest that the mutant HBV clones comprising various drug-resistant mutations could latently exist even in the liver of NA treatment-naïve cases.

### Expansion of drug-resistant HBV clones harboring M240VI mutations in response to NA administration

To clarify the risk of latent expansion of drug-resistant mutations due to NA treatment, we next examined the early dynamic changes of the prevalence of M204VI mutants in the serum of treatment-naïve patients in response to entecavir treatment. Ultra-deep sequencing provided a mean 40,791- and 38,823-fold coverage of readings, which were mapped to the M204VI nucleotide position at the YMDD sites of each reference sequence in patients before and after entecavir treatment.

Five of 14 (35.7%) patients harbored the M204VI mutations prior to entecavir treatment. Although the serum HBV DNA levels were significantly reduced in response to entecavir in all cases, the M204VI mutant clones were detected in 9 cases (64.3%) after entecavir administration (Table 5). Notably, one patient (Serum #3) who harbored the M240VI mutant clones at baseline had a relatively large expansion of drug-resistant clones among the total viral population in a time-dependent manner in response to entecavir treatment (Table 5). Similarly, M240VI mutant clones became detectable after entecavir administration in four patients (Serum #1, #7, #12, #13) that harbored no resistant mutants at baseline (Table 5). We found no correlation between the degree of the increase in the relative prevalence of M204VI mutant clones and that of the reduction in serum HBV DNA levels. Although only a limited number of patients exhibited a substantial increase in M204VI mutant clones after administration of anti-viral therapy, our findings might suggest that entecavir treatment latently causes selective survival of drug-resistant mutants in treatment naïve patients with chronic HBV infection.

**Table 5:** The prevalence of M204VI mutation at YMDD site in patients before and after entecavir administration.

	Entecavir treatment				
	Before		After		
	Prevalence of the mutated clones		Prevalence of the mutated clones		Period of NA treatment
Serum #3	222/32,238	(0.7%)	2,284/23,791	(9.6%)	2w
Serum #2	401/34,041	(1.2%)	266/25,301	(1.1%)	24w
Serum #5	521/48,723	(1.1%)	245/25,521	(1.0%)	56w
Serum #8	748/65,573	(1.1%)	336/28,702	(1.2%)	48w
Serum #9	312/30,599	(1.0%)	169/14,172	(1.2%)	56w
Serum #1	9/22,843	(-)	2,839/34,162	(8.3%)	8w
Serum #7	26/65,564	(-)	923/66,458	(1.4%)	4w
Serum #12	91/65,616	(-)	258/27,958	(0.9%)	24w
Serum #13	11/23,209	(-)	206/64,747	(0.3%)	32w
Serum #4	3/7,923	(-)	39/65,575	(-)	12w
Serum #6	52/65,582	(-)	77/55,273	(-)	16w
Serum #10	38/22,522	(-)	8/21,053	(-)	8w
Serum #11	47/43,853	(-)	5/16,520	(-)	16w
Serum #14	42/42,784	(-)	40/36,668	(-)	12w

Mutation frequency (%): the ratio of total mutant clones to total aligned coverage at each nucleotide sites.

(-): mutant clones less than 0.3% among total clones at each nucleotide sites.

## Discussion

Direct population sequencing is the most common method for detecting viral mutations [29]. Conventional sequencing techniques, however, are not efficient for evaluating large amounts of genetic information of the viruses. Newly developed ultra-deep sequencing technology have revolutionized genomic analyses, allowing for studies of the dynamics of viral quasispecies as well as rare genetic variants of the viruses that cannot be detected using standard direct population sequencing techniques [30], [31]. The sensitivity of ultra-deep sequencing analysis is primarily limited by errors introduced during PCR amplification and the sequencing reaction, thus it is a challenge to distinguish rare variants from sequencing artifacts. In the present study, we optimized the ultra-deep sequencing with a multiplex-tagging method and reproducibly detected variants within HBV quasispecies that were as rare as 0.3%. Based on this ultra-deep sequencing platform, we determined the abundant genetic heterogeneity of HBV at the intra- and inter-individual levels.

Because of its ability to handle abundant viral genome information, ultra-deep sequencing allowed us to evaluate low-abundant virus variants of patients with chronic HBV infection in detail. It is widely accepted that HBe seroconversion is highly associated with the emergence of G1896A pre-C and/or A1762T and G1764A core promoter mutant clones [7]–[9]. Unexpectedly, however, our results showed a diverse range of G1896A frequency (0–99.9%) in HBeAg-negative subjects and a high prevalence of core promoter mutations, irrespective of HBe serostatus. Consistent with our observation, previous studies utilizing conventional sequencing methods reported that the frequency of the G1896A pre-C mutant ranged from 12% to 85% [32]. All but one patient (Liver #10) showing a predominance of A1762T and G1764A were infected with genotype C, while patient#10 was infected with genotype B. Because A1762T and G1764A are reported to be significantly more frequent in genotype C [33], the difference in the prevalence of A1762T and G1764A in our study might be a reflection of the viral HBV genotype rather than HBe serostatus. Further investigation of the actual prevalence of these mutations and the elucidation of other unknown mutations involved in HBe seroconversion are necessary for a better understanding of the underlying mechanisms of HBe seroconversion.

One thing to be noted is that the majority of the chronic-NA cases had extremely low levels of the G1896A pre-C mutant in their liver tissues, even though those cases were serologically positive for anti-HBe and negative for HBeAg. Moreover, entecavir administration significantly reduced the proportion of the G1896A pre-C mutant in the serum of the majority of patients irrespective of their HBeAg serostatus, while the G1896A pre-C mutant clones were detectable in a substantial proportion before treatment in all cases. These findings suggest that the G1896A pre-C mutant have higher sensitivity to NA than the wild-type viruses. Consistent with this hypothesis, several previous studies reported that NA is effective against acute or fulminant hepatitis caused by possible infection with the G1896A pre-C mutant [34], [35]. Based on these findings, early administration of NA might be an effective strategy for treating patients with active hepatitis infected predominantly with the G1896A pre-C mutant.

Ultra-deep sequencing has a relatively higher sensitivity than conventional direct population sequencing and is thus useful for detecting drug-resistant mutations not detected by standard sequencing [20], [21]. Recently, we revealed that drug-resistant mutants were widely present in treatment-naïve HCV-infected patients, suggesting a putative risk for the expansion of resistant clones to anti-viral therapy [19]. Here, we demonstrated that various drug-resistant HBV variants are present in a proportion of chronically HBV-infected, NA-naïve patients. Several studies using ultra-deep sequencing provided evidence that naturally-occurring drug-resistant mutations are detectable in treatment-naïve individuals with human immunodeficiency virus-1 infection [30], [36], [37]. Consistent with the cases of human immunodeficiency virus-1 infection, a few studies detected minor variants resistant to NA in the plasma of treatment-naïve patients with chronic HBV infection [20], [21]. It remains unclear, however, whether these minor drug-resistant mutations have clinical significance. Our observation of the relative expansion of viral clones with the M204VI mutation during entecavir therapy in some cases indicates the possibility that preexisting minor mutants might provide resistance against NA through the selection of dominant mutant clones. Future studies with a larger cohort size are required to clarify the clinical implications of the latently existing low-abundant drug-resistant mutations.

The current ultra-deep parallel sequencing technology has limitations in the analyses of viral quasispecies. First, because the massively-parallel ultra-deep sequencing platform is based on a multitude of short reads, it is difficult to evaluate the association between nucleotide sites mapped to different genome regions in a single viral clone. Indeed, potential mutational linkages between the pre-C and reverse transcriptase regions were difficult to elucidate due to the short read length of the shotgun sequencing approach. Second, accurate analysis of highly polymorphic viral clones by ultra-deep sequencing is also difficult because the identification of mutations depends strongly on the mapping to the reference genome sequences.

In conclusion, we demonstrated that the majority of patients positive for anti-HBe and negative for HBeAg lacked the predominant infection of the G1896A pre-C mutant in the presence of NA treatment, suggesting that the G1896A pre-C mutant have increased sensitivity to NA therapy compared with wild-type HBV. We also revealed that drug-resistant mutants are widely present, even in the liver of treatment-naïve HBV-infected patients, suggesting that the preexisting low-abundant mutant clones might provide the opportunity to develop drug resistance against NA through the selection of dominant mutations. Further analyses utilizing both novel and conventional sequencing technologies are necessary to understand the significance and clinical relevance of the viral mutations in the pathophysiology of various clinical settings in association with HBV infection.

## Supporting Information

Figure S1 **Comparison of the viral complexity between the liver and serum of the same individual.** Shannon entropy values throughout the whole viral genome of the liver and serum of the representative two cases are shown. (upper two panels, case #11; lower two panels, case #14). preC-C: pre-core∼core, preS: pre-surface, P: polymerase.
(TIF)

Table S1 **The oligonucleotide primers for amplifying HBV sequences in each clinical specimen.**
(DOCX)

Table S2 **Error frequency of Ultra-deep sequencing for the expression plasmid encoding wild-type genotype C HBV genome sequences by the three control experiments.**
(DOCX)

Table S3 **The sensitivity and accuracy of detecting the low abundant minor clones in association with the different coverage numbers.**
(DOCX)
