# Supplementary figures and images for: Dynamics of Hepatitis B Virus Quasispecies in Association with Nucleos(t)ide Analogue Treatment Determined by Ultra-Deep Sequencing
Source: PLoS One. 2012 Apr 16;7(4):e35052. doi: 10.1371/journal.pone.0035052 (PMC3327662; doi:10.1371/journal.pone.0035052)

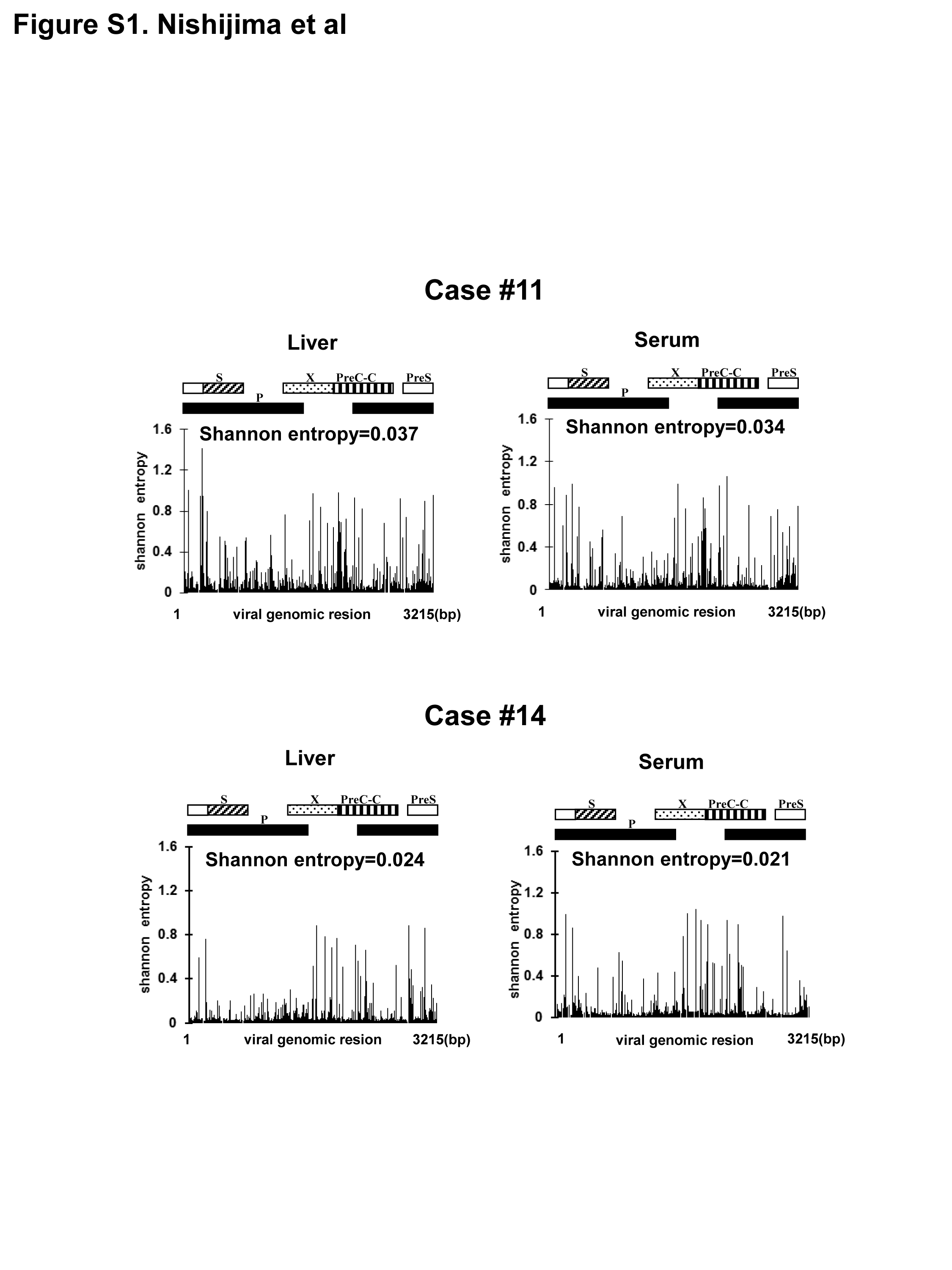

Supplement: Figure S1 — Comparison of the viral complexity between the liver and serum of the same individual. Shannon entropy values throughout the whole viral genome of the liver and serum of the representative two cases are shown. (upper two panels, case #11; lower two panels, case #14). preC-C: pre-core∼core, preS: pre-surface, P: polymerase. (TIF) [file pone.0035052.s001.tif]
