# Supplementary material for: Dynamics of Hepatitis B Virus Quasispecies in Association with Nucleos(t)ide Analogue Treatment Determined by Ultra-Deep Sequencing
Source: PLoS One. 2012 Apr 16;7(4):e35052. doi: 10.1371/journal.pone.0035052 (PMC3327662; doi:10.1371/journal.pone.0035052)
Supplement: Table S1 — The oligonucleotide primers for amplifying HBV sequences in each clinical specimen. (DOCX) [file pone.0035052.s002.docx]

**Table S1. The oligonucleotide primers for amplifying HBV sequences in each clinical specimen.**

| Primer | 5’-Positon* | Sequence | Forward / Reverse | Usage |
| --- | --- | --- | --- | --- |
| 169_F | 169 | ATCAGGATTCCTAGGACCC | Forward | PCR for amplicon 1 |
| 2847_R | 2847 | GCTGTAGCTCTTGTTCCCAAG | Reverse | PCR for amplicon 1 |
| 685_F | 685 | GCCATTTGTTCAGTGGTTCG | Forward | PCR for amplicon 2 |
| 443_R | 443 | GAACCAACAAGAAGATGAGGC | Reverse | PCR for amplicon 2 |
| 1896WT_F | 1877 | CTGTGCCTTGGGTGGCTTTG | Forward | quantitative real-time PCR |
| 1896MT_F | 1877 | CTGTGCCTTGGGTGGCTTTA | Forward | quantitative real-time PCR |
| 2037_R | 2037 | TCCGGAGACTCTAAGGCCTC | Reverse | quantitative real-time PCR |

*All the positions were relative to HBV genotype C sequence (GenBank AB033550.1)
