# Supplementary material for: Dynamics of Hepatitis B Virus Quasispecies in Association with Nucleos(t)ide Analogue Treatment Determined by Ultra-Deep Sequencing
Source: PLoS One. 2012 Apr 16;7(4):e35052. doi: 10.1371/journal.pone.0035052 (PMC3327662; doi:10.1371/journal.pone.0035052)
Supplement: Table S2 — Error frequency of Ultra-deep sequencing for the expression plasmid encoding wild-type genotype C HBV genome sequences by the three control experiments. (DOCX) [file pone.0035052.s003.docx]

**Table S2. Error frequency of Ultra-deep sequencing for the expression plasmid encoding wild type genotype C HBV genome sequences by the three control experiments.**

|  | Run #1 | Run #2 | Run #3 |
| --- | --- | --- | --- |
| Total aligned reads | 75,507 | 47,846 | 109,638 |
| Total aligned nucleotides | 4,832,416 | 3,062,114 | 7,016,830 |
| Average coverage | 37,172 | 23,555 | 53,976 |
| Type of errors |  |  |  |
| mismatches | 1,185 | 684 | 3,676 |
| deletions | 63 | 32 | 107 |
| insertions | 4 | 0 | 0 |
| Overall error rate (%) | 0.026 | 0.023 | 0.054 |
| Range of error rate(%) | 0-0.09 | 0-0.13 | 0-0.13 |
