# Supplementary material for: Dynamics of Hepatitis B Virus Quasispecies in Association with Nucleos(t)ide Analogue Treatment Determined by Ultra-Deep Sequencing
Source: PLoS One. 2012 Apr 16;7(4):e35052. doi: 10.1371/journal.pone.0035052 (PMC3327662; doi:10.1371/journal.pone.0035052)
Supplement: Table S3 — The sensitivity and accuracy of detecting the low abundant minor clones in association with the different coverage numbers. (DOCX) [file pone.0035052.s004.docx]

**Table S3. The sensitivity and accuracy of detecting the low abundant minor clones in association with the different coverage numbers.**

| Coverage | Major allele (%)* | Minor allele (%)** | Error rate (%)^†^ |
| --- | --- | --- | --- |
| 478 | 99.79 | **0.21** | 0 |
| 860 | 99.88 | **0.12** | 0 |
| 1644 | 99.82 | **0.12** | 0.06 |
| 6177 | 99.72 | **0.24** | 0.04 |
| 9750 | 99.84 | **0.11** | 0.05 |
| 20550 | 99.85 | **0.12** | 0.03 |
| 65687 | 99.77 | **0.23** | 0 |

*Major allele (%): the frequency of the wild-type plasmid detected by ultra-deep sequence.

**Minor allele (%): the frequency of the mutant plasmid detected by ultra-deep sequence.

^†^Error (%): the frequency of the nucleotide except for original viral sequences detected by ultra-deep sequence.
